# Cerebellar ataxias: β‐III spectrin's interactions suggest common pathogenic pathways

**DOI:** 10.1113/JP271195

**Published:** 2016-04-24

**Authors:** Emma Perkins, Daumante Suminaite, Mandy Jackson

**Affiliations:** ^1^ Centre for Integrative PhysiologyUniversity of EdinburghHugh Robson Building, George SquareEdinburghEH8 9XDUK

## Abstract

Spinocerebellar ataxias (SCAs) are a genetically heterogeneous group of disorders all characterised by postural abnormalities, motor deficits and cerebellar degeneration. Animal and *in vitro* models have revealed β‐III spectrin, a cytoskeletal protein present throughout the soma and dendritic tree of cerebellar Purkinje cells, to be required for the maintenance of dendritic architecture and for the trafficking and/or stabilisation of several membrane proteins: ankyrin‐R, cell adhesion molecules, metabotropic glutamate receptor‐1 (mGluR1), voltage‐gated sodium channels (Na_v_) and glutamate transporters. This scaffold of interactions connects β‐III spectrin to a wide variety of proteins implicated in the pathology of many SCAs. Heterozygous mutations in the gene encoding β‐III spectrin (*SPTBN2*) underlie SCA type‐5 whereas homozygous mutations cause spectrin associated autosomal recessive ataxia type‐1 (SPARCA1), an infantile form of ataxia with cognitive impairment. Loss‐of β‐III spectrin function appears to underpin cerebellar dysfunction and degeneration in both diseases resulting in thinner dendrites, excessive dendritic protrusion with loss of planarity, reduced resurgent sodium currents and abnormal glutamatergic neurotransmission. The initial physiological consequences are a decrease in spontaneous activity and excessive excitation, likely to be offsetting each other, but eventually hyperexcitability gives rise to dark cell degeneration and reduced cerebellar output. Similar molecular mechanisms have been implicated for SCA1, 2, 3, 7, 13, 14, 19, 22, 27 and 28, highlighting alterations to intrinsic Purkinje cell activity, dendritic architecture and glutamatergic transmission as possible common mechanisms downstream of various loss‐of‐function primary genetic defects. A key question for future research is whether similar mechanisms underlie progressive cerebellar decline in normal ageing.

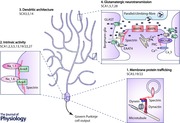

## Introduction

The cerebellum is essential for maintaining postural control and coordination of voluntary muscle movement (Manto, 2008). Purkinje cells, the principal neurons and sole output of the cerebellar cortex, exhibit autonomous high‐frequency repetitive firing in addition to receiving input from inhibitory interneurons and two excitatory fibres, climbing and parallel fibres. Purkinje cells integrate the information and transmit timing signals essential for motor coordination in the form of inhibitory inputs to the deep cerebellar nuclei (DCN). The DCN, in turn, communicate with various parts of the nervous system controlling movement. Alterations to Purkinje cell and consequently DCN activity (Shakkottai *et al*. 2004) are therefore sufficient to induce ataxia, a phenotype characterised by gait disturbances, postural instability and motor incoordination.

Autosomal dominant spinocerebellar ataxias (SCAs), a heterogeneous group of inherited neurodegenerative disorders, are a major cause of cerebellar ataxia. Their prevalence in several populations can be as high as 5–6 in 100,000 (Ruano *et al*. 2014), similar to that of Huntington's and motor neuron disease. All SCAs can be characterised by postural abnormalities, progressive motor incoordination and cerebellar degeneration, but a number of subtypes can also be associated with additional neurological features such as cognitive impairment. To date 40 different genomic loci, numbered in order of discovery, have been associated with SCAs and the genes involved, along with the responsible mutations, have been identified for 26 SCA subtypes.

The first genetic defects to be identified as associating with SCA1, 2, 3, 6, 7 and 17 were coding for CAG repeat expansions, leading to proteins with abnormally long poly‐glutamine (polyQ) tracts (Orr *et al*. 1993; Kawaguchi *et al*. 1994; Imbert *et al*. 1996; Pulst *et al*. 1996; Sanpei *et al*. 1996; David *et al*. 1997; Zhuchenko *et al*. 1997; Nakamura *et al*. 2001). Together they account for more than half of all SCA cases, with SCA3 being the most common (Ruano *et al*. 2014). Subsequently, non‐coding CAG repeats (Holmes *et al*. 1999; Koob *et al*. 1999), non‐CAG repeat expansions (Matsuura *et al*. 2000; Sato *et al*. 2009; Kobayashi *et al*. 2011) and, more recently, conventional mutations have been found to underlie different SCA subtypes (Table 1). This latter category is ever expanding, due to the advent of whole‐exome sequencing, and although conventional mutations are often associated with rarer forms of SCA, they have provided substantial insight into the physiological defects underlying ataxia.

**Table 1:** Conventional mutations and molecular mechanisms underlying spinocerebellar ataxias

SCA subtype	Gene	Protein	Normal function	Disease mechanism	DNA mutations	References
SCA5/SPARCA1	SPTBN2	β‐III spectrin	Membrane support, protein trafficking_,_ stabilisation	Loss‐of‐function, DN	ID, MM, FM	Ikeda *et al*. 2006; Lise *et al*. 2012
SCA11	TTBK2	Tau tubulin kinase 2	Protein phosphorylation, ciliogenesis	Loss‐of‐function, DN	FM	Houlden *et al*. 2007; Goetz *et al*. 2012
SCA13	KCNC3	K_v_3.3	Neuronal excitability, K^+^ homeostasis	Loss‐of‐function, DN	MM	Waters *et al*. 2006; Irie *et al*. 2014
SCA14	PRKCG	Protein kinase C (PKC)	Protein phosphorylation	Unknown	MM, D	Chen *et al*. 2003
SCA15/16/29	ITPR1	Inositol 1,4,5‐trisphosphate receptor type 1	Calcium homeostasis	Loss‐of‐function	MM, D	van de Leemput *et al*. 2007; Iwaki *et al*. 2008; Huang *et al*. 2012
SCA19/22	KCND3	K_v_4.3	Neuronal excitability, K^+^ homeostasis	Loss‐of‐function, DN	ID, MM	Duarri et al. 2012; Lee *et al*. 2012
SCA23	PDYN	Prodynorphin	Opioid signalling	Unknown	MM, D	Bakalkin *et al*. 2010; Jezierska *et al*. 2013
SCA26	EEF2	Eukaryotic translation elongation factor 2	Protein translation	Loss‐of‐function	MM	Hekman *et al*. 2012
SCA27/episodic ataxia	FGF14	Fibroblast growth factor 14	Modulation of Na_v_ channels, signal transduction	Loss‐of‐function	MM, D	van Swieten *et al*. 2003; Brusse *et al*. 2006; Shakkottai *et al*. 2009; Choquet *et al*. 2015
SCA28	AFG3L2	ATPase family gene 3‐like 2	ATP‐dependent protease activity	Haplo‐insufficiency	MM, FM	Maltecca *et al*. 2009: Di Bella *et al*. 2010; Musova *et al*. 2014
SCA35	TGM6	Transglutaminase	Modification of glutamine residues	Loss‐of‐function	ID, MM	Wang *et al*. 2010: Guo *et al*. 2014
SCA40	CCDC88C	Coiled‐coil domain containing protein 88C	JNK signalling	Gain‐of‐function	MM	Tsoi *et al*. 2014

DN, dominant‐negative; ID, in‐frame deletion; MM, missense mutation; FM, frame‐shift mutation; D, large deletion.

The focus of this review is genetic analyses and use of experimental models to elucidate the pathogenesis of spinocerebellar ataxia type 5 (SCA5). Evidence will be presented demonstrating how changes in Purkinje cell intrinsic excitability, dendritic architecture and synaptic function, observed in mouse models of SCA5, have contributed to our understanding of cerebellar dysfunction in SCA5 and how similar physiological defects may be associated with other SCAs.

## Heterozygous mutations in *SPTBN2* gene give rise to spinocerebellar ataxia type 5

**Figure 1:**
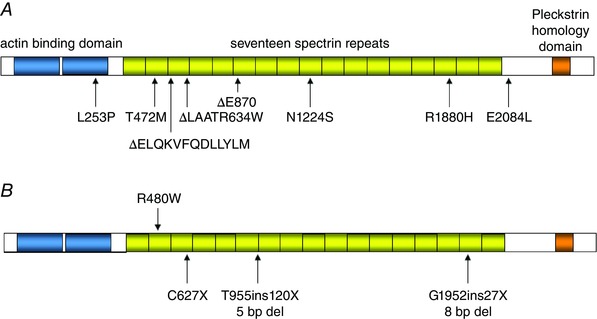
**Structure of β‐III spectrin and localisation of mutations** *A*, β‐III spectrin comprises an N‐terminal actin binding domain, 17 spectrin repeats and a pleckstrin homology (PH) domain at the C‐terminus, which can facilitate interaction with the lipid bilayer. Mutations associated with SCA5 are indicated: missense mutation (L253P) within second calponin‐homology (CH) domain, German family (Ikeda *et al*. 2006); missense mutation (T472M) within second spectrin repeat, Norwegian family (Cho & Fogel, 2013); 39 bp in‐frame deletion (Δ39) resulting in 13‐amino‐acid deletion (E532_M544del) within third spectrin repeat, Lincoln pedigree (Ikeda *et al*. 2006); 15 bp in‐frame deletion (L629_R634) and missense mutation (R634W), French family (Ikeda *et al*. 2006); 3 bp in‐frame deletion resulting in glutamic acid deletion (E870del) in sixth spectrin repeat, Japanese family (Wang *et al*. 2014). Co‐segregation of mutations N1224S, R1880H and E2804L with ataxia is uncertain (Zühlke *et al*. 2007). *B*, location of mutations associated with infantile cerebellar ataxia: heterozygous R480W mutation identified in two cases of infantile ataxia with global developmental delay (Jacob *et al*. 2012; Parolin Schnekenberg *et al*. 2015); homozygous stop codon (C627X) within third spectrin repeat, UK family (Lise *et al*. 2012); homozygous 5 bp frameshift deletion (T955ins120X) in spectrin repeat 6, Egyptian family (Elsayed *et al*. 2014). Homozygous 8 bp frameshift deletion (G1952ins27X) in spectrin repeat 16, Beagle puppies (Forman *et al*. 2012).

The genetic locus for SCA5 was mapped to the centromeric region of the long arm of chromosome 11 (11q13) using a large kindred descended from the paternal grandparents of United States President Abraham Lincoln (Ranum *et al*. 1994). Later a French (Stevanin *et al*. 1999) and a German (Burk *et al*. 2004) pedigree with a similarly mild form of SCA were linked to the same chromosomal region. Mutations were subsequently identified in the *SPTBN2* gene encoding β‐III spectrin (Fig. 1 *A*; Ikeda *et al*. 2006), which is found throughout the cell body and dendritic tree of Purkinje cells (Jackson *et al*. 2001).

The initial symptoms of SCA5 are disturbance of gait, incoordination of limbs, abnormal eye movements and slurred speech. Yet age of onset is variable within families, starting between the second and seventh decade. Typically there is no reduction in lifespan, possibly due to the lack of bulbar paralysis which in other SCAs appears to result in a poorer ability to fight recurrent pneumonia (Zoghbi, 1991) and patients remain ambulatory for several decades. Pathologically severe atrophy of the cerebellum is observed with magnetic resonance imaging (MRI) and autopsy examination shows significant Purkinje cell loss, shrinkage of the molecular layer, mild loss of granular neurons and empty basket fibres (Ikeda *et al*. 2006).

## Infantile ataxia and cognitive impairment associated with mutations in *SPTBN2*

Homozygous mutations in *SPTBN2* were recently found in two families with both cerebellar ataxia from childhood and cognitive impairment (Fig. 1 *B*), classifying an allelic condition, spectrin associated autosomal recessive cerebellar ataxia type 1 (SPARCA1) (Lise *et al*. 2012; Elsayed *et al*. 2014). A complete loss‐of β‐III spectrin function is thus implicated in motor and cognitive deficits from birth. However, a novel heterozygous mutation (R480W) has also been reported in a patient exhibiting infantile onset and global developmental delay (Jacob *et al*. 2012). It may be that in this case there is an undetected mutation in *trans* or an environmental modifier resulting in a much earlier and more severe phenotype than other β‐III spectrin heterozygous mutations. Alternatively residue R480, within spectrin repeat 2, could be of particular structural importance. Notably, the same heterozygous R480W mutation was recently identified in a child originally given a working diagnosis of ataxia cerebral palsy (Parolin Schnekenberg *et al*. 2015), strengthening the evidence that mutation R480W is more deleterious than other heterozygous *SPTBN2* mutations.

Variability in presentation has similarly been observed for mutations in other SCA‐associated loci. Mutations in the inositol 1,4,5‐trisphosphate receptor type 1 gene (*ITPR1*) have been reported in families with late onset SCA15 (van de Leemput *et al*. 2007), early‐onset SCA29 (Iwaki *et al*. 2008) and sporadic infantile‐onset cerebellar ataxia (Huang *et al*. 2012). Mutations in the genes *KCNC3* (Waters *et al*. 2006; Parolin Schnekenberg *et al*. 2015) and *FGF14* (Coebergh *et al*. 2014; Planes *et al*. 2015) have also been associated with variable phenotypes. The molecular reason(s) for differences in timing of onset remain unknown, but the clinical characteristics of patients with early‐onset disease are generally non‐progressive ataxia, motor developmental delay and mild cognitive deficits. Understanding the molecular mechanisms whereby early‐onset cases of ataxia are associated with cognitive impairment could help address whether the cerebellum plays a developmental role in cognition or if the deficits are non‐cerebellar in origin.

## Loss‐of protein function in cerebellar pathogenesis

Animal models have proved instrumental in elucidating the pathogenesis of SCA. To date three different SCA5 mouse models have been generated and analysed for signs of motor incoordination and cerebellar degeneration in relation to disrupted β‐III spectrin function (Table 2). Two mouse models were created by gene disruption, one by exon trapping (Spnb3^−/−^; Stankewich *et al*. 2010) and the other by targeted recombination (β‐III^−/−^; Perkins *et al*. 2010). The third is a conditional transgenic model which utilises the tetracycline transactivator protein (tTA) under the control of the Purkinje cell specific promoter Pcp2 to specifically drive wild‐type or Δ39 β‐III spectrin in cerebellar Purkinje cells (Armbrust *et al*. 2014). All three models exhibit motor impairment but only the β‐III^−/−^ mouse model recapitulates the progressive motor deficits and Purkinje cell loss observed in SCA5 patients (Perkins *et al*. 2010). The β‐III^−/−^ mouse model in particular has implicated neuronal dysfunction in the early motor phenotype with gait and coordination deficits evident prior to any cerebellar degeneration. It, together with the identification of the allelic condition SPARCA1, has also provided insights into the molecular dominance in SCA5. Results suggest the disease is due to loss‐of‐function but not due to β‐III spectrin haploinsufficiency as no phenotype is observed in 2‐year‐old heterozygous (β‐III^+/−^) mice (Clarkson *et al*. 2010) or elderly heterozygous SPARCA1 carriers (Lise *et al*. 2012). Instead SCA5 pathogenesis is likely to occur when β‐III spectrin function falls below 50% of wild‐type level due to interference by mutant protein.

**Table 2:** Molecular, anatomical and behavioural characteristics of mouse lines generated to model SCA5

	Spnb3^−/−^	β‐III^−/−^	Pcp2‐tTA^+/−^/TRE‐SPΔ39^+/−^
	(Stankewich *et al*. 2010)	(Perkins *et al*. 2010)	(Armbrust *et al*. 2014)
Protein expressed	Truncated β‐III spectrin, terminating at start of spectrin repeat 14	β‐III spectrin lacking exons 2–6 (actin binding domain)	Full complement of mouse β‐lll spectrin. Human Δ39β‐III spectrin
Protein distribution	Mislocalised to axon initial segment	Normal somatodendritic distribution	Normal somatodendritic distribution
Motor defects	Slightly poorer performance on rotarod at 8 months of age. Not progressive	Progressive ataxia with mild motor impairment at 3 weeks of age. By 6 months of age unable to stay on rotarod at 3 r.p.m.	Mild impairment on rotarod at 26 weeks of age. Later time points not analysed
Cerebellar degeneration	Thinning of molecular layer at 8 months. No PC loss at 1.5 years	ML thinning and PC loss visible by 6 months, greater at 1 year	Thinning of ML at 80 weeks. No PC loss
Unexpected phenotype	Myoclonic seizures. Muscle weakness	None	None

Loss‐of protein functions due to dominant‐negative effects have also been reported for a number of other SCA subtypes (Table 1). Knock‐out animals therefore have the potential to mirror disease phenotypes of autosomal dominant SCAs more faithfully than transgenic models. However, full characterisation for the presence of truncated proteins that could either abrogate loss‐of protein function or confer an aberrant function is essential, as is functional analysis to validate models as true knockouts. Creation of representative transgenic models also requires, in order to avoid gene dosage effects on phenotype, detailed information regarding the stability and expression level of mutant proteins and the minimum level of wild‐type protein critical for normal function. This is highlighted by work using *Drosophila* models of SCA5 where progressive neurodegeneration was observed in the *Drosophila* eye when human β‐III spectrin containing either the German (L253P) or American (Δ39) mutation was ectopically overexpressed (Lorenzo *et al*. 2010). The phenotype, however, was milder in flies hemizygous for L253P spectrin, with these flies expressing significantly lower levels of transgenic protein than flies expressing Δ39 spectrin. The phenotype could be enhanced by increasing L253P spectrin expression through creation of homozygous flies. Similarly posterior paralysis was only observed in larvae expressing a single copy of human Δ39 spectrin when one copy of the endogenous β‐spectrin gene was silenced. Hopefully with the advent of new gene targeting technologies (TALENs, Crispr/Cas9) the creation of knock‐in models will be greatly facilitated, circumventing caveats surrounding level of transgene expression for loss‐of‐function models.

## Common molecular mechanisms underpinning cerebellar dysfunction in SCAs

Analyses of the different animal models and a number of *in vitro* studies have implicated various molecular mechanisms in the cerebellar dysfunction associated with SCA5. In particular they converge on alterations to glutamatergic transmission and Purkinje cell excitability, arising from a role for β‐III spectrin in membrane protein trafficking, localisation and stabilisation. Disruption to these same physiological processes is evident in models of other SCAs, highlighting the possible convergence of common mechanisms in cerebellar ataxia.

### Disruption in membrane protein trafficking, localisation and/or stabilisation

**Figure 2:**
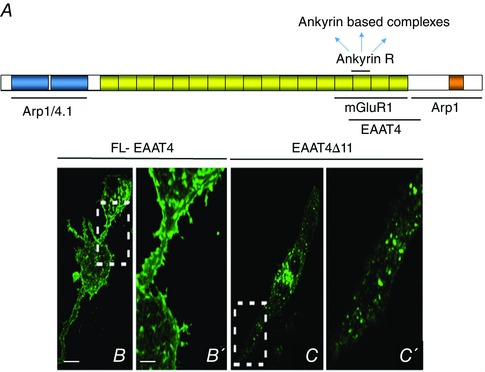
**Defective protein trafficking when β‐III spectrin's scaffold of protein interactions is disrupted** *A*, schematic diagram depicting identified interacting partners of β‐III spectrin: Arp1, ankyrin R, mGluR1 and EAAT4. *B* and *B*′, full‐length (FL) EAAT4 located at cell membrane and in spine‐like protrusions when overexpressed in Neuro2a cells. *C* and *C*′, EAAT4 lacking β‐III spectrin's interacting domain within terminal 11 amino acids (EAAT4Δ11) is located peri‐nuclearly and in large intracellular vesicles. Scale bar: *B* and *C*, 20 μm; *B*′ and *C*′, 10 μm.

The actin binding domain and C‐terminus of β‐III spectrin (Fig. 2 *A*) were both shown in a yeast two‐hybrid assay to directly bind to Arp 1 (Holleran *et al*. 2001), a subunit of the dynactin complex which mediates the association of vesicular cargo with the microtubule motor dynein (Karki *et al*. 2000). Further support for β‐III spectrin's role in protein vesicular trafficking is the co‐purification from rat brain vesicles with Arp1 and dynein (Holleran *et al*. 2001) and disruption to axonal transport in flies expressing either the American or German mutant β‐III spectrin, with enhancement of these transport abnormalities in dynein and dynactin loss‐of‐function mutants (Lorenzo *et al*. 2010). Both β‐III spectrin knockout (Spnb3^−/−^ and β‐III^−/−^) mouse lines also exhibit dilatation of endoplasmic reticulum and alterations to Golgi structure indicating an important function of β‐III spectrin in the trafficking of membrane proteins (Perkins *et al*. 2010; Stankewich *et al*. 2010).

It has been shown that β‐III spectrin interacts directly with the carboxy‐terminus of EAAT4 (Fig. 2 *A*; Jackson *et al*. 2001), the glutamate transporter found in Purkinje cell soma and dendrites (Yamada *et al*. 1996; Dehnes *et al*. 1998). The interaction stabilises EAAT4 at the plasma membrane, resulting in an increase in cell surface expression and enhanced glutamate uptake (Jackson *et al*. 2001). In contrast, mutant Δ39 β‐III spectrin failed to restrict the lateral mobility of EAAT4 in HEK 293 cells indicating an inability to properly anchor EAAT4 at the plasma membrane (Ikeda *et al*. 2006). Co‐expression of mutant L253P β‐III spectrin in HEK 293 cells was also found to disrupt post‐Golgi trafficking of EAAT4, with normal cell surface expression only attainable when cells were incubated at a lower temperature (Clarkson *et al*. 2010). Similarly, EAAT4 lacking the terminal 11 amino acids and hence the β‐III spectrin binding motif, remains peri‐nuclear or in large intracellular vesicular structures when expressed in Neuro2a (Fig. 2 *C*). In contrast full length EAAT4 is present at the cell surface in Neuro2a cells enriched in spine‐like protrusions (Fig. 2 *B*). Together these *in vitro* findings support an important role for β‐III spectrin in the cellular trafficking and stabilisation of EAAT4 at the plasma membrane. Importantly, reduced EAAT4 levels were observed in young β‐III^−/−^ and Spnb3^−/−^ mice with EAAT4 accumulating in the cell soma and dendritic shafts (Perkins *et al*. 2010; Stankewich *et al*. 2010; Gao *et al*. 2011), similar to SCA5 autopsy tissue (Ikeda *et al*. 2006). Loss of EAAT4 and β‐III spectrin prior to onset of symptoms was also reported in a transgenic mouse model of SCA1 that specifically expresses in Purkinje cells human ataxin‐1 with a pathological (82) polyglutamine repeat length (ATXN1^Q82^) (Lin *et al*. 2000; Serra *et al*. 2004). More direct evidence that EAAT4 loss is causal in cerebellar dysfunction comes from recent analyses of EAAT4 knockout animals which were found to exhibit motor deficits prior to cerebellar degeneration (unpublished data). No early loss of EAAT4 was observed in the SCA5 transgenic model, but this may be a consequence of the low expression level of Δ39 β‐III spectrin transgene (Armbrust *et al*. 2014).

Expression and stability of other membrane proteins have also been reported to be dependent on β spectrin. A decrease in two cell adhesion molecules, neuroglian and Fasciclin II (Fas II), was observed in *Drosophila* lacking presynaptic β spectrin (Pielage *et al*. 2005) and an altered distribution of Fas II was seen in flies expressing SCA5 mutant spectrin (Lorenzo *et al*. 2010). Recently β‐III spectrin repeats 14–16 were shown to interact with the metabotropic glutamate receptor mGluR1α and TIRF microscopy revealed wild‐type but not mutant Δ39 β‐III spectrin could increase the stability of mGluR1α–green fluorescent protein (GFP) at the plasma membrane (Armbrust *et al*. 2014). The recruitment and maintenance of ankyrin R at the plasma membrane of Purkinje cell dendrites also seems to depend on β‐III spectrin (Clarkson *et al*. 2014), and further direct evidence for the importance of this interaction in SCA pathogenesis comes from normoblastosis (*nb*/*nb*) mice, deficient in erythroid ankyrin, which develop abnormal gait, tremor and 50% loss of Purkinje cells by the age of 6 months (Peters *et al*. 1991).

β‐III spectrin is not believed to be expressed in Bergmann glia, but loss of the glial glutamate transporter GLAST (EAAT1 in humans) was observed in both Spnb3^−/−^(Stankewich *et al*. 2010) and β‐III^−/−^ (Perkins *et al*. 2010) mouse lines, indicating an indirect effect on glial membrane protein stability, possibly arising from disruption to cell–cell adhesion and signalling molecules. In β‐III^−/−^ mice the decrease in GLAST has been implicated in the progression of motor deficits (Perkins *et al*. 2010 and unpublished data) and correlations between decreased GLAST expression and Purkinje cell loss were also reported for transgenic ATXN1^Q82^ SCA1 mice (Cvetanovic, 2015) and mice expressing, only in Bergmann glia, mutant ataxin 7 protein (Custer *et al*. 2006). Understanding the mechanisms that underpin loss of GLAST will be important as these may highlight potential strategies for mitigating disease progression. There is also evidence that loss of EAAT1 is a primary cause of ataxia with mutations in *SLC1A3*, the gene encoding excitatory amino acid transporter 1, giving rise to episodic ataxia (Jen *et al*. 2005; de Vries *et al*. 2009), further supporting the idea that loss of GLAST is more than a simple consequence of a different primary genetic defect.

### Changes to intrinsic Purkinje cell activity

**Figure 3:**
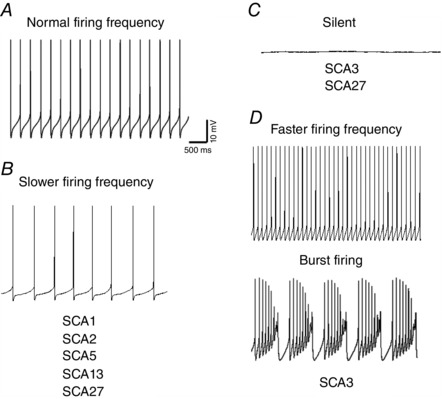
**Intrinsic activity of Purkinje cells altered in mouse models of SCA** *A*, representative trace of a current‐clamp *in vitro* slice recording for a spontaneously firing Purkinje cell. *B*, slower firing rates compared to controls observed in models of SCA1, 2, 5, 13 and 27. *C*, quiescent Purkinje cells identified in SCA3 and 27 models, about one‐half and 80% of cells, respectively. *D*, faster tonic firing rate and burst firing observed in remaining SCA3 tg/– Purkinje cells.

**Figure 4:**
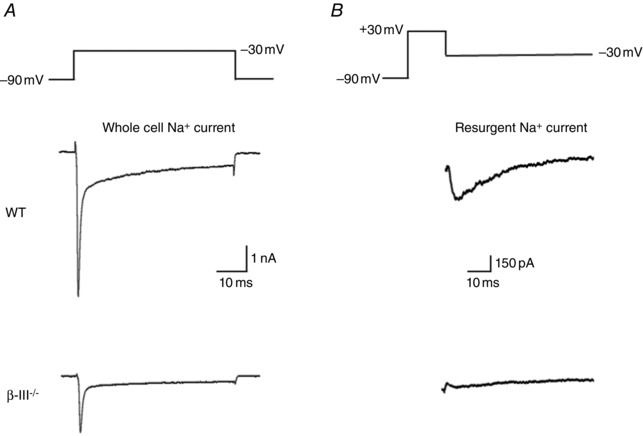
**Smaller sodium currents are likely to underpin reduced intrinsic activity** *A*, representative current traces from acutely dissociated Purkinje cells elicited with a step depolarisation to −30 mV from a holding potential of −90 mV. *B*, resurgent sodium currents evoked using a 20 ms step to +30 mV, from a holding potential of –90 mV, followed by repolarisation to −30 mV. Top traces are from wild‐type Purkinje cells and bottom traces from β‐III^−/−^ Purkinje cells showing reduced whole cell Na^+^ currents and absence of resurgent currents.

A key component of Purkinje cell output is their intrinsic activity, which has been found in *in vitro* electrophysiological recordings to be altered in various SCA models (Table 3 and Fig. 3). It is governed by specific ion channels and in particular Na_v_1.1 and 1.6 channels, the two dominant Na_v_ channels in cerebellar Purkinje neurons, both of which possess a resurgent sodium current (Raman & Bean, 1997; Raman *et al*. 1997; Khaliq *et al*. 2003; Kalume *et al*. 2007). Sodium channel dysfunction was observed in the β‐III^−/−^ mouse model of SCA5 prior to cerebellar atrophy with smaller whole‐cell and resurgent sodium currents recorded from dissociated Purkinje cells isolated from P16–P20 β‐III^−/−^ mice (Fig. 4; Perkins *et al*. 2010). This is consistent with the slower rate of Purkinje cell tonic firing observed in cerebellar slices from 3‐week‐old β‐III^−/−^ mice (Fig. 3 *B*; Perkins *et al*. 2010) and may well be a consequence of decreased Na_v_1.1 and 1.6 stability in the absence of a β‐III spectrin/ankyrin R anchor (Clarkson *et al*. 2014). Functional *in vitro* analyses of two SCA5‐associated mutant β‐III spectrin proteins (L253P and R634W) also showed diminished effects in enhancing sodium currents compared to wild‐type β‐III spectrin with reduced ankyrin R and Na_v_ channel levels associated with this effect (Clarkson *et al*. 2014). Together the data indicate that reduced Purkinje cell intrinsic activity due to a decreased stability of the β‐III spectrin/ankyrin‐R/Na_v_ complex is likely to be a critical component of SCA5 pathogenesis. The heterozygous R480W mutation associated with infantile ataxia was found to have a similar effect to the L253P and R634W mutants (Parolin Schnekenberg *et al*. 2015) and so additional structural and expression studies are required to resolve whether the change of arginine to tryptophan at reside 480 is more physiologically deleterious than the other heterozygous mutations so far characterised.

**Table 3:** Common Purkinje cell intrinsic activity defects in models of SCA

SCA subtype	Physiological deficit	Molecular mechanism
SCA1	Reduced intrinsic firing frequency. Irregular plateau potential	Increased K_v_4.3 surface expression
SCA2	Reduced intrinsic firing frequency	Increased Ca^2+^ release from intracellular stores
SCA3	Purkinje cells either silent through depolarisation block or display faster intrinsic firing rate/burst firing	Increased K_v_3 channel inactivation
SCA5	Reduced intrinsic firing frequency	Decrease in whole‐cell and resurgent sodium current
SCA13	Reduced intrinsic firing frequency. Broader action potential	Decrease in K_v_3.3 activity
SCA27	Reduced intrinsic firing frequency	Decrease in resurgent sodium
	or Purkinje cells silent	current

A similar decrease in Purkinje cell excitability resulting from sodium channel dysfunction was reported for SCA27 pathogenesis, the genetic causes of which are two loss‐of‐function mutations, a point mutation (F145S) or a frameshift mutation (Asp163fsX12), in the intracellular fibroblast growth factor 14 (iFGF14) gene (Wang *et al*. 2002; van Swieten *et al*. 2003; Dalski *et al*. 2005). iFGFs bind directly to the cytoplasmic C‐terminal domains of Na_v_ channel α subunits, with wild‐type FGF14 increasing Na_v_ current densities in hippocampal neurones (Lou *et al*. 2005), whereas peak sodium currents were reduced in cells expressing SCA27 disease associated mutation FGF14^F145S^ (Laezza *et al*. 2007). It is thought that FGF14 functions as an oligomeric protein and FGF14^F145S^ acts as a dominant negative disrupting the association of wild‐type FGF14 and Na_v_ channel α subunits. Such a role for loss‐of‐Nav channel modulation and decreased neuronal excitability in SCA27 pathogenesis is supported by the absence *in vitro* of spontaneous activity in Purkinje cells both from FGF14‐null mice (Shakkottai *et al*. 2009), which exhibit a very similar phenotype to that of SCA27 patients and following *in vivo* iFGF14 knock‐down studies (Bosch *et al*. 2015). Reduced Na_v_ resurgent sodium current amplitudes and spontaneous firing rates were also observed following acute knockdown of iFGF14 in cultured Purkinje cells (Yan *et al*. 2014). A key feature of SCA27 pathogenesis appears to be enhanced Na_v_ channel inactivation and loss of resurgent current downstream of FGF14 loss‐of‐function.

K_v_3 channels are also indispensable for high‐frequency intrinsic firing as they exhibit fast activation and deactivation kinetics. Missense mutations (R420H, R423H and F448L) in the gene encoding human K_v_3.3 (*KCNC3*) give rise to SCA13 (Waters *et al*. 2006; Irie *et al*. 2014). Since all K_v_ channels are formed by the assembly of four subunits, K_v_3.3 channels in SCA13 are likely to consist of WT and mutant subunits with normal function being disrupted in a dominant‐negative manner. This is supported by the fact smaller outward currents, broadened action potential wave‐forms and a reduced firing frequency are observed in cultured Purkinje cells expressing mouse K_v_3.3–R424H (Irie *et al*. 2014), the equivalent of human R423H, similar to *in vitro* recordings from K_v_3.3 knockout mice (Hurlock *et al*. 2008). The resulting delay in Purkinje cell repolarisation is thought to instigate cell death by increasing Ca^2+^ influx through excessive activation of Ca_v_ channels.

Mutations giving rise to SCA19/22 were also recently identified in another K_v_ subunit, K_v_4.3, and are predicted to reduce cerebellar output, similar to SCA13, as they impair trafficking of the channel to the plasma membrane and/or reduce channel activity (Duarri *et al*. 2012; Lee *et al*. 2012). In contrast, accumulation of K_v_4.3 channels at the cell surface was observed in the SCA1 ATXN1^Q82^ mouse model (Hourez *et al*. 2011). Five‐week‐old ATXN1^Q82^ mice displayed impaired motor performance and reduced *in vitro* firing frequencies, with a proportion of cells showing an irregular plateau potential, but no cell atrophy or death at this age. Both the firing frequency and the motor performance were restored by treatment with DiAP, a potassium channel blocker. The molecular mechanisms underlying the increase in K_v_4.3 surface expression and mode of DiAP action are not yet fully understood, although the former is suggested to be linked to reduced K_v_ internalisation due to smaller glutamate receptor‐mediated postsynaptic currents.

Alterations to Purkinje cell firing prior to signs of neurodegeneration were also observed in *in vitro* slice recordings for mouse models of SCA3 with 84 glutamine repeats in the *ATXN3* gene (Shakkottai *et al*. 2011) and SCA2 with 127 glutamine repeats in human ataxin‐2 cDNA (Hansen *et al*. 2013). About one half of the SCA3 tg/− Purkinje cells were found to be silent, with a depolarised membrane potential and the others either displayed a faster firing rate than wild‐type or exhibited repetitive bursts demonstrating increased excitability (Fig. 3 *D*). Depolarisation block, through increased K_v_3 current inactivation, was reported to give rise to the loss of repetitive firing, but how mutant ataxin‐3 alters K_v_3 channel kinetics is not known. One possibility is that it affects the post‐translational modification of potassium channels. In the case of *ATXN2* ^Q127^ mice a progressive slowing in the firing rate was observed with age but additional analyses are required to determine the molecular mechanisms responsible.

### Altered Purkinje cell dendritic architecture

**Figure 5:**
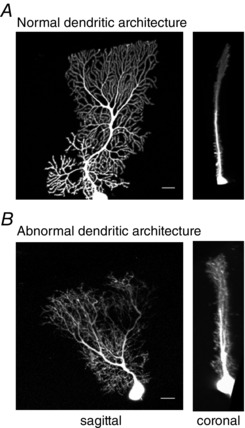
**Early morphological changes to Purkinje cell dendritic architecture implicated in neuronal dysfunction** Individual Purkinje cells from 6‐week‐old WT (*A*) and β‐III^−/−^ (*B*) animals filled with Alexa Fluor 568 and imaged at Nyquist sampling rates (Scale bar, 20 μm). Thinner, disordered branching and greater dendritic protrusion in coronal plane evident in Purkinje cells lacking β‐III spectrin. Morphological changes can result in changes to resting membrane potential and number and/or specificity of synaptic inputs.

Cerebellar output is also influenced by the integration of excitatory and inhibitory inputs that modulate intrinsic Purkinje cell activity (Hausser & Clark, 1997). Since the elaborate monoplanar Purkinje cell dendritic tree determines both the number and type of input and how the synaptic signals decay as they propagate towards the soma (Rall, 1977; Hausser *et al*. 2000; Gulledge *et al*. 2005), alterations to dendritic morphology will affect Purkinje cell output. In young β‐III^−/−^ mice the Purkinje cell dendritic trees were found to be disordered and no longer planar, and dendrites were thinner (Fig. 5; Gao *et al*. 2011). Membrane properties are affected by dendritic diameter (Rall, 1977) and so aberrant activation of low voltage‐gated calcium channels and excessive calcium entry, a potential consequence of thinner dendrites, may contribute to neuronal dysfunction. Loss of planarity prior to cell death can also alter synaptic inputs through interdigitation of neighbouring dendritic trees. Multiple climbing fibre (CF) innervation can arise via CF transverse branches (Miyazaki & Watanabe, 2011) and disruption to the specificity of granule cell input can occur through ascending axons making synaptic contact with more than one Purkinje cell and/or parallel fibres making additional contacts with the same Purkinje cell (Napper & Harvey, 1988).

Changes to Purkinje cell dendritic architecture were also reported for Purkinje cells in an SCA3 mouse model expressing N‐terminally truncated *ATXN3^Q69^* protein (Konno *et al*. 2014) and for a cellular model of SCA14 (Seki *et al*. 2009), which is caused by missense mutations in the *PRKCG* gene encoding protein kinase Cγ (PKCγ). Expression of either mutant S119P or G128D PKCγ in cultured Purkinje cells resulted in reduced dendritic area, dendrite diameter and spine density (Seki *et al*. 2009). Conventional PKCs require Ca^2+^ for activation and can regulate actin cytoskeleton dynamics through modulation of adducin and recruitment of spectrin to the ends of actin filaments (Matsuoka *et al*. 1998). Morphological changes could be a common feature in a number of SCAs due to dysregulated Ca^2+^ homeostasis and downstream effects on PKC activity and cytoskeletal dynamics.

### Defects in glutamatergic neurotransmission

**Figure 6:**
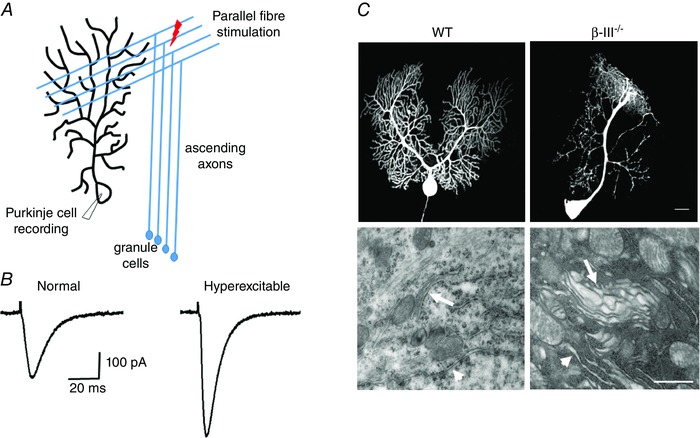
**Excessive glutamatergic innervation and AMPA receptor‐mediated delayed excitotoxicity in Purkinje cell degeneration** *A*, parallel fibre‐mediated Purkinje cell postsynaptic excitatory currents (PF‐EPSCs) measured by applying a stimulus within the molecular layer. *B*, greater excitability in Purkinje cells from 3‐ to 6‐week‐old β‐III^−/−^ animals compared to WT animals shown by larger PF‐mediated EPSC amplitude. *C*, individually filled Purkinje cells reveal dendritic degeneration and ultrastructural analysis using transmission electron microscopy demonstrates dark cell degeneration in Purkinje cells from 8‐month‐old β‐III^−/−^ but not WT animals. Dilatation of endoplasmic reticulum (ER), ER denuded of ribosomes (arrowhead) and fragmentation of Golgi (arrow) are all features of AMPA receptor‐elicited delayed excitotoxicity. Scale bar: top panel, 20 μm; bottom panel, 0.5 μm).

Purkinje cells, due to the large amount of afferent glutamatergic input they receive from both parallel and climbing fibres through activation of ionotropic AMPA and metabotropic (mGluRs) receptors, are especially vulnerable to glutamate‐mediated excitotoxicity and elevations in intracellular calcium (Fig. 6). Aberrant glutamatergic neurotransmission has been observed in two of the SCA5 mouse models. Enhanced parallel fibre‐mediated excitatory postsynaptic currents (PF‐EPSCs) were recorded from young β‐III^−/−^ mice compared to wild‐type mice (Fig. 6 *A* and *B*; Perkins *et al*. 2010). Although initially the increase in excitability is thought to partially offset the reduced spontaneous activity, the excessive activation of AMPA receptors would appear to be ultimately detrimental, with Purkinje cells from 8‐month and older animals found to exhibit dendritic degeneration, undergo dark cell degeneration (Fig. 6C) and have reduced *in vivo* output (Perkins *et al*. 2010).

The glutamatergic defect detected in the SCA5 transgenic model is reduced mGluR1 activity following parallel fibre stimulation, due to mislocalisation but not loss of mGluR1 protein (Armbrust *et al*. 2014). Impairment of mGluR1 signalling was also reported in the *ATXN3^Q69^* mouse model (Konno *et al*. 2014). A similar loss of mGluR1 signalling may not be detected in the two SCA5 knock‐out models as unlike the transgenic animals they both exhibit a loss of EAAT4 protein. EAAT4 modulates the activation of perisynaptic mGluRs, with high EAAT4 expressing Purkinje cells exhibiting very little mGluR activity (Wadiche & Jahr, 2005). Loss of EAAT4 in Spn3^−/−^ and β‐III^−/−^, as well as ATXN1^Q82^ mice may therefore result in excessive mGluR activation and downstream dysregulated calcium homeostasis. This would be similar to recent findings in a mouse model of SCA28, which is haploinsufficient for Afg3l2 and displays dark cell degeneration of Purkinje cells (Maltecca *et al*. 2009). Reducing mGluR1 activity was found to decrease Ca^2+^ levels in Afg3l2^+/−^ Purkinje cells and reverse the ataxic phenotype (Maltecca *et al*. 2015) indicating attenuating mGluR1 signalling may possess therapeutic promise.

The generation and analyses of various SCA mouse models has revealed possible common physiological deficits downstream of different primary genetic defects. Alterations to intrinsic firing through either direct or indirect effects on ion channels critical for maintaining fast repetitive Purkinje cell firing have been observed in the early stages of cerebellar ataxia in a number of SCA models. The majority of these studies have utilised *in vitro* slice recordings and so in the future it may be informative to assess the cerebellar output in awake animals. Alterations to Purkinje cell Ca^2+^ homeostasis, in a number of instances arising from altered glutamatergic transmission, are another mechanism common across SCAs which could contribute to dysregulated PKC activity, cytoskeletal alterations, aberrant dendritic architecture and ultimately cell death.

## Summary

Cerebellar ataxias can all be characterised by the same clinical features (postural abnormalities, progressive motor incoordination and cerebellar degeneration) highlighting that although the underlying primary genetic defects differ, the downstream molecular mechanisms are likely to converge, with the ultimate effect of altered cerebellar output being common to all. Studies outlined in this review have identified alterations to intrinsic Purkinje cell excitability, dendritic morphology and glutamatergic transmission, arising from disruption to membrane protein trafficking, localisation and stabilisation, as factors pertinent to altered cerebellar output following loss of β‐III spectrin function. The necessity for orchestration of protein networks in normal cerebellar physiology is exemplified by the disruption of β‐III spectrin function and demonstrates how it is possible that defects in different components of a protein network can instigate the same pathogenic pathway.

Given that motor and cognitive decline are associated with normal ageing, a key question is whether changes to the spectrin submembranous meshwork and key membrane proteins might underpin age‐related changes in performance. It has been reported that a progressive increase in α‐II spectrin proteolysis (Cai *et al*. 2012; Hwang *et al*. 2012), a calcium‐dependent process linked to Purkinje cell toxicity (Mansouri *et al*. 2007), is associated with age. Dilatation of the endoplasmic reticulum and degeneration of the Golgi apparatus (Dlugos, 2005), reduction in glutamate transporters and functional glutamate uptake associated with mGluR activation (Potier *et al*. 2010; Brothers *et al*. 2013; Pereira *et al*. 2014) as well as changes to the distribution of Na_v_ channels (Chung *et al*. 2003) have also all been reported in aged rodents. Dysregulation of glutamatergic neurotransmission and Purkinje cell excitability may therefore be an important feature of age‐related cerebellar decline. Similarly cerebellar abnormalities have also been linked to the pathophysiology of Alzheimer's disease (Sjöbeck & Englund, 2001; Mavroudis *et al*. 2013), schizophrenia (Andreasen & Pierson, 2008), autism (Courchesne *et al*. 1994; Palmen *et al*. 2004; Whitney *et al*. 2008) and other cognitive and neuropsychiatric disorders (Schmahmann & Sherman, 1998; Konarski *et al*. 2005; Alalade *et al*. 2011; Stoodley & Stein 2011). The ongoing challenge for researchers will be to decipher subtle changes in the morphological and molecular integrity of the cerebellar cortex that underpin Purkinje cell dysfunction both in early stages of various neurological disorders and in normal ageing.

## Additional information

### Competing interests

None of the authors has any conflicts of interest.

### Author contributions

All authors have approved the final version of the manuscript and agree to be accountable for all aspects of the work. All persons designated as authors qualify for authorship, and all those who qualify for authorship are listed.

### Funding

The Wellcome Trust (EP & MJ) and Ataxia UK/RS MacDonald Charitable Trust (DS).
